# Editorial Board Members’ Collection Series: “Neuroinflammation”

**DOI:** 10.3390/ijms262110473

**Published:** 2025-10-28

**Authors:** Savina Apolloni, Sushruta Koppula

**Affiliations:** 1 Department of Biology, Tor Vergata University of Rome, 00133 Rome, Italy; 2 Department of Biotechnology, College of Biomedical and Health Sciences, Konkuk University, Chungju-Si 27478, Chungcheongbuk-do, Republic of Korea

More than 600 brain diseases have been identified [1], and neuroinflammation is implicated in nearly all of them. Complex and disorder-specific changes in the immune response underlie the pathogenesis of primary neurological and neuropsychiatric conditions from pediatric to geriatric disorders. These include neurodegenerative diseases (e.g., Parkinson’s disease, PD; Alzheimer’s disease, AD), developmental disorders (e.g., cerebral palsy), cerebrovascular conditions (e.g., ischemic stroke, IS; vascular dementia), traumatic injuries (e.g., traumatic brain injury, TBI), convulsive disorders (e.g., epilepsy), infectious diseases (e.g., AIDS-related dementia), and brain tumors.

Neuroinflammation encompasses infiltrating immune cells into the central nervous system (CNS) and activating resident microglia and astrocytes [2]. It contributes not only to disease onset and progression but also to plasticity, recovery, and therapeutic responses [3]. While neuroinflammation can have protective roles, countering threats and promoting lesion repair, its detrimental consequences often outweigh these beneficial effects, as persistent inflammatory cascades can drive neurodegeneration [4].

This Special Issue “Editorial Board Members’ Collection Series: Neuroinflammation” brings together original research, reviews, and translational protocols that span the full spectrum from molecular mechanisms to clinical applications. The first group of papers focuses on experimental and molecular studies, addressing mechanisms, novel compounds, and nutraceutical interventions, while the second group focuses on clinical and translational perspectives, focusing on human disease contexts, systematic reviews, and innovative therapeutic strategies.

Bjedov et al. [5] explored the effects of a novel de novo synthesized derivative of cholic bile acid, SB140, an immunomodulatory activator of nuclear factor-erythroid two related factor 2 (Nrf2), on CNS immune cells. Their findings provide compelling evidence for the anti-inflammatory potential of this small molecule. Specifically, SB140 activated Nrf2 signaling in microglia, reducing the production of key pro-inflammatory mediators, including interleukin (IL)-6, tumor necrosis factor (TNF), and nitric oxide. Furthermore, SB140 suppressed both proliferation and effector cytokine release in encephalitogenic T cells, notably IL-17 and interferon (IFN)-γ. These dual effects on innate and adaptive immune cells position SB140 as a promising candidate for therapeutic modulation of neuroinflammation. Importantly, this study underscores the therapeutic promise of novel small-molecule Nrf2 activators in modulating pathological immune responses within the CNS, particularly in neuroinflammatory conditions such as multiple sclerosis, where impaired Nrf2 activation is a hallmark feature.

In another contribution, Zhang et al. investigated the role of C15orf39, a novel MAPK1 substrate, in regulating microglial inflammatory responses [6]. Using human HMC3 microglia stimulated with lipopolysaccharide (LPS) and IFN-γ, the authors demonstrated that C15orf39 expression was downregulated with increased production of pro-inflammatory cytokines, including IL-6 and TNFα. Functional studies revealed that overexpression of C15orf39 suppressed IL-6 and TNFα expression, whereas its knockdown promoted their release under inflammatory conditions. Mechanistically, C15orf39 was shown to interact with the cytoplasmic protein arginine methyltransferase 2 (PRMT2). This interaction attenuated NF-κB activation through the PRMT2-IκBα axis, reducing inflammatory mediators’ transcription. Intriguingly, under pro-inflammatory stimulation, NF-κBp65 itself was activated and, in turn, suppressed C15orf39 promoter activity, thereby overriding the inhibitory effect of the C15orf39-PRMT2-IκBα pathway. These findings identify C15orf39 as a suppressive regulator of microglial activation and highlight its potential as a therapeutic target in CNS disorders characterized by chronic neuroinflammation, including Alzheimer’s disease.

Finally, the study by Andraka and colleagues examined the effects of krill oil (KO) supplementation, a marine-based, long-chain omega-3, consisting of polyunsaturated fatty acids (PUFAs), in counteracting the effects of cognitive aging and a high-fat diet on neuroinflammation and hippocampal plasticity of aged rats [7]. Despite the strong theoretical basis, given KO’s known anti-inflammatory and antioxidant properties, and its enriched composition of bioactive compounds, the current dose of KO provided for this study did not enhance many of the brain parameters measured, including spatial memory, cytokine concentrations, microglia count, synaptic density, or neurogenesis. The study’s rigorous negative result contributes essential methodological insight, serving as a baseline for fine-tuning future interventions, particularly regarding early-life initiation of supplementation, extended duration, and dosage optimization. The study underscores the necessity for refined experimental designs to understand how and under what conditions PUFA-rich supplements might benefit aging and obesity-induced neural decline.

These studies illustrate how diverse experimental strategies offer valuable insights into the molecular and cellular mechanisms of neuroinflammation. By highlighting both positive and null findings, these works collectively advance our understanding of how neuroinflammatory processes can be modulated, and they point toward promising avenues for therapeutic intervention in neuroimmune and neurodegenerative disorders.

In the second group of articles, the authors majorly focused on the clinical and translational perspectives on neuroinflammation. The authors emphasized the crucial role of neuroinflammation and neuroplasticity in various condition including TBI, IS and PD, highlighting how inflammatory mediators impair recovery depending on their regulation. Targeting microglial, IL-1 family members and associated cytokines, and neurotrophic signaling emerges as a promising therapeutic approach in TBI, IS, and PD. The articles also highlight integration of biomarkers such as pro- and anti-inflammatory markers, BDNF, exosomes, could improves prognosis and personalized treatment approaches. Overall, combining anti-inflammatory interventions along with neurotrophic factor holds strong potential to enhance neurorestoration and improve behavioral outcomes in patients.

The review by Calderone et al. [3] established an impressive correlation between neuro-inflammation and neuroplasticity in TBI recovery. Chronic inflammation during TBI, often driven by mechanisms including activation of microglia and NLRP3 inflammasome, leukotriene signaling, and enhanced levels of pro-inflammatory mediators, which can suppress synaptic plasticity particularly in regions like the hippocampus, cortex, and white matter, impacting learning-memory, emotional and motor function [8,9,10,11]. Authors have demonstrated that targeting neuro-inflammation could be beneficial in controlling neuroplasticity alterations associated with TBI. Interventions targeting microglial dynamics, such as microglial replacement could delay chronic neuroinflammation and improves behavioral and cognitive outcomes after TBI, could play central role in recovery [12]. Furthermore, interventions such as MK-886, 5-lipoxygenase-activating protein (FLAP) inhibitors and PLX5622, CSF1R antagonists can restore neuronal health are based on microglia-driven pathways [13,14]. In parallel, sleep fragmentation, as a behavioral factor exacerbate neuroinflammation through activation of HPA-axis dysregulation, altering glucocorticoid receptor activity, further impairing recovery, highlighting the multifactorial influences on neuromodulatory outcomes after TBI [15].

Clinical and preclinical studies have highlighted implication of biomarkers in monitoring shift over neuromodulation and impact of neuro-inflammation on synaptic plasticity, thus making them critical in planning personalized treatment and improves recovery in TBI. The authors discussed the advancement in biomarkers research such as S100β, YKL-40, procalcitonin, GFAP, C-reactive protein, and tau, along with immerging biomarkers SAA1 and TLR4, correlate strongly with structural and functional outcomes, as evidenced by imaging studies, cognitive and motor assessments [14,16,17] (Figure 1). Collectively, these reviews point toward a dual approach for TBI management, involving use of anti-inflammatory strategies along with early detection and monitoring of biomarkers in order to personalize interventions to optimize neuroplasticity and cognitive outcome.

The review by Matys et al. [18] provides comprehensive understanding about involvement damage associated molecular patterns (DAMPs) along with IL-1 family members in ischemic stroke (Figure 2). The IL-1 family, includes both pro-inflammatory members (IL-1α, IL-1β, IL-18, IL-36) and anti-inflammatory members (IL-1Ra, IL-33, IL-37, IL-38, IL-36Ra) markers, plays crucial role upsetting delicate balance between promoting and suppressing inflammation, and thus provides possible biomarker and helps in developing novel therapeutic interventions targeting IL-1 family. The authors discussed the activation of IL-1α and IL-1β during early ischemia, which act on IL-type 1 receptor (IL-1R1) and triggers downstream signaling pathways such as MyD88-IRAK, TRAF6-NF-κB and MAPKs, results in upregulation of adhesion molecules, cytokines, chemokines, matrix metalloproteinases (MMPs), recruiting immune cells.

On the regulatory side, anti-inflammatory members including IL-1Ra, IL-37, IL-38, and IL-33 limits damage by dampening inflammation, reducing infarct size, influencing repair and improving neurological outcomes. Among the members, IL-1Ra, anakinra (a recombinant human IL-1Ra) and IL-33 show strong neuroprotection (Figure 3), while IL-37 and IL-38 inhibit pro-inflammatory cytokine signaling [19,20]. Additionally, IL-33 promotes neurorestoration via ST2 induced Treg and ILC2 activation [21], whereas elevated levels of sST2, a natural IL-33 inhibitor, have been correlated with stroke severity [22]. Furthermore, authors have discussed involvement of genetic association in influencing the role of IL-1 family cytokines in IS. Studies have shown that polymorphisms in the IL-1β gene, such as C allele of the IL-1β rs1143627 and T allele at position-511 variants, may increase susceptibility to IS [23]. Likewise, polymorphisms in the IL-33/ST2, including rs10435816, rs7025417, rs11792633, and rs7044343, are associated with reduced risk of atherosclerotic IS. In conclusion, given the dual nature of inflammation, the balance between activities of IL-1 family cytokines seems critical in determining severity of a brain damage and patient recovery. However, many gaps remain to be addressed including exact timing and localization of different IL-1 family member expressions; association between comorbidities; and clinical potential of targeting IL-1 family member. The review suggests that IL-1 family cytokines could serve as useful biomarkers for diagnosis and monitory treatment.

In another review article by Riccitelli et al. [24], the authors highlighted the potential of high-frequency repetitive transcranial magnetic stimulation (rTMS) in enhancing BDNF expression and neurocognitive functions in PD. The authors discussed the potential of BDNF, a synaptic marker for neuroplasticity, in the survival and function of dopaminergic neurons in the striatum and substantia nigra, a region involved in PD [25,26]. Extracellular vesicles (EVs), particularly exosomes, which carry BDNF, have been linked with motor severity and functional impairments in PD, thus making them an ideal candidate for investigating changes associated with disease [27]. Considering the reported potential of HF-rTMS in neurodegenerative conditions such as AD, authors have applied HF-rTMS targeting of the dorsolateral prefrontal cortex (DLPFC) to enhance BDNF expression in PD. They performed a randomized, sham-controlled trial in PD patients with mild cognitive impairment. HF-rTMS was applied bilaterally to the DLPFC over 20 sessions (45 min each, one per day) in 4 weeks. Plasma and EV-derived BDNF along with executive functions were measured at baseline (T0), post-treatment (T1), and 8-week follow-up (T2). Data are analyzed using mixed-factorial ANOVA and regression to measure treatment response and cognition correlations.

The study involves the use of different groups, which allowed the author to differentiate the effect of placebo with treatment; additionally, the use of MRI-guided neuronavigation enhanced the precision of DLPFC targeting, thus improving the validity and enhancing the reproducibility of the study. The study also measured the plasma BDNF and exosomal plasma-derived BDNF to capture the neurotrophic effects of rTMS. HF-rTMS found to modulate executive functions in PD patients by enhancing BDNF-mediated synaptic neuroplasticity in the DLPFC, along with dopamine release in fronto-striatal circuits. The study proposes exosomal BDNF as a novel biomarker involved in regulating neuroplasticity changes in brain. Furthermore, clinical implications of these findings highlight HF-rTMS as a promising therapeutic approach for addressing both cognitive and motor challenges in PD.

In conclusion, the articles presented in this Special Issue reflect the multifaceted aspects of neuroinflammation research. Experimental investigations provide mechanistic insights and identify novel modulatory strategies, while clinical and translational studies connect these findings to potential applications in patients with neurological disorders. To develop effective interventions, future research will require integrating these perspectives, combining molecular approaches, nutraceuticals, and neuromodulatory techniques with clinical insights.

## Figures and Tables

**Figure 1:**
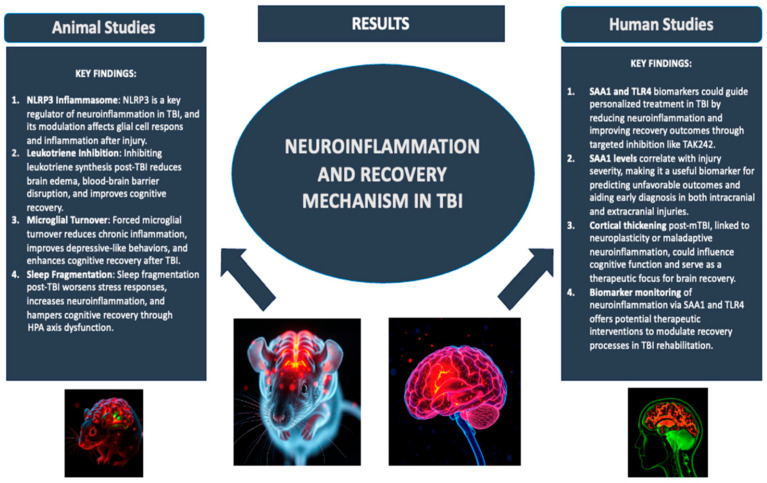
Neuroinflammation and recovery mechanism in TBI. Adapted from [3].

**Figure 2:**
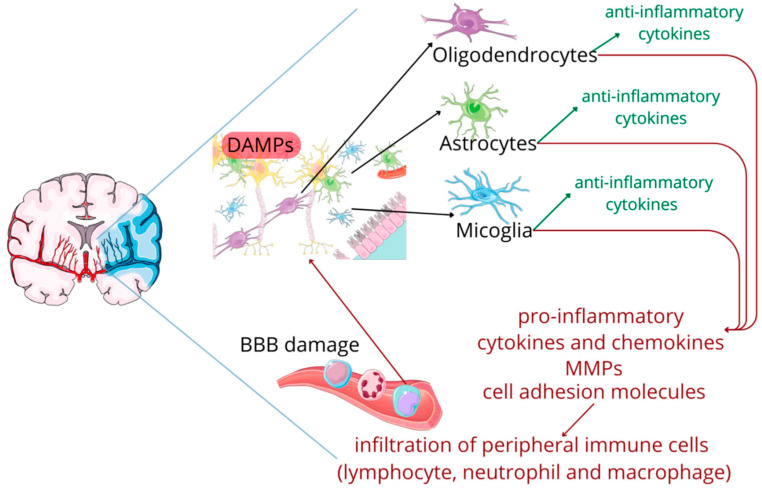
The cascade following an ischemic stroke. Adapted from [18].

**Figure 3:**
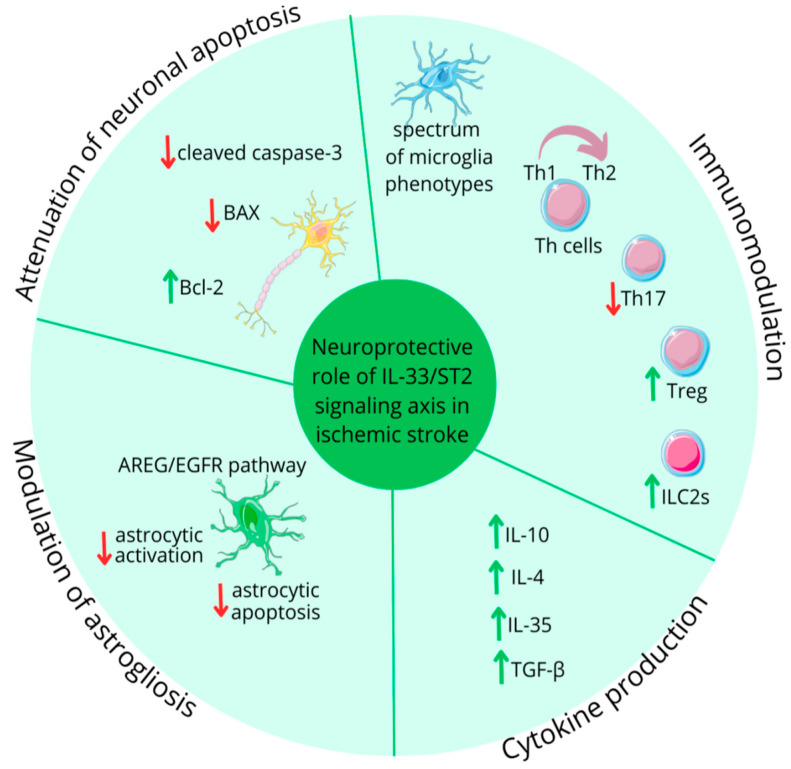
IL-33 role in ischemic from ‘Expanding Role of Interleukin-1 Family Cytokines in Acute Ischemic Stroke’. Adapted from [18].
